# Workplace gender composition and psychological distress: the importance of the psychosocial work environment

**DOI:** 10.1186/1471-2458-14-241

**Published:** 2014-03-10

**Authors:** Sofia Elwér, Klara Johansson, Anne Hammarström

**Affiliations:** 1 Department of Public Health and Clinical Medicine, Umeå University, SE90187, Umeå, Sweden

**Keywords:** Workplace, Gender composition, Gender, Psychological distress, Psychosocial work environment, Gender segregation

## Abstract

**Background:**

Health consequences of the gender segregated labour market have previously been demonstrated in the light of gender composition of occupations and workplaces, with somewhat mixed results. Associations between the gender composition and health status have been suggested to be shaped by the psychosocial work environment. The present study aims to analyse how workplace gender composition is related to psychological distress and to explore the importance of the psychosocial work environment for psychological distress at workplaces with different gender compositions.

**Methods:**

The study population consisted of participants from the Northern Swedish Cohort with a registered workplace in 2007 when the participants were 42 years old (N = 795). Questionnaire data were supplemented with register data on the gender composition of the participants’ workplaces divided into three groups: workplaces with more women, mixed workplaces, and workplaces with more men. Associations between psychological distress and gender composition were analysed with multivariate logistic regression analysis adjusting for socioeconomic position, previous psychological distress, psychosocial work environment factors and gender. Logistic regression analyses (including interaction terms for gender composition and each work environment factor) were also used to assess differential associations between psychosocial work factor and psychological distress according to gender composition.

**Results:**

Working at workplaces with a mixed gender composition was related to a higher likelihood of psychological distress compared to workplaces with more men, after adjustments for socioeconomic position, psychological distress at age 21, psychosocial work environment factors and gender. Psychosocial work environment factors did not explain the association between gender composition and psychological distress.

**Conclusions:**

The association between gender composition and psychological distress cannot be explained by differences in the perception of the psychosocial work environment and thus the work environment hypothesis is not supported. Workplaces with a mixed gender composition needs further research attention to explain the negative development of psychological distress during working life for both women and men at these workplaces.

## Background

How the gender segregated labour market is related to the gendered distribution of psychological distress and somatic illness is an emerging research field [1]. One of the most salient features of the gender segregated labour market is the gender segregation of occupations and workplaces into men’s work and women’s work. Few employees, in Sweden only 13 per cent, work in occupations where women and men are equally represented (with between 40–60 per cent women and men) [2]. Gender segregation can be identified and studied at the macro level of society in the gender segregated labour market, and all the way down to the micro division of labour in work tasks [3]. How gender segregation of work is related to ill-health has mainly been studied in relation to gender composition of *occupations*[4-6]. However, the gender composition of the occupation does not always reflect the gender composition of the actual work site as several occupations often form a work group. The degree of contact among workers within the same occupation at a workplace can vary, making both occupations and workplace gender composition valid units of analysis. The few studies that have focused on *workplaces* have analysed gender composition in relation to sickness absence and self-rated health [7-10], whereas mental health remains unexplored. Although mental illness accounts for a substantial part of the sickness absence in Sweden, especially for women [11], there is also a need for studies that focus on the mental ill- health aspects of the gender composition. The present study seeks to explore how the gender composition of a workplace is related to mental ill-health and the importance of the psychosocial work environment for that relation.

There are several models that seek to explain how the gender composition of a workplace can be related to different health outcomes. Based on Kanter’s theory of “tokens”, researchers have suggested that being a small minority at the workplace could be extra harmful for health status [12,13]. However, this theory has not been supported empirically [7,14]. For women, the highest levels of sickness absence have been found at workplaces with a majority of women [7,8]. For men, one study showed no differences in sickness absence in relation to workplace gender composition [7], and another study showed poorer self-rated health among men at workplaces with a majority of women [9]. In addition to the token hypothesis, a work environment hypothesis has been suggested that states that the health consequences of the psychosocial work environment are contextual and therefore dependent on the gender composition of the workplace [10]. Previous research has shown, for example, that high strain jobs at male-dominated workplaces are associated with higher odds of sickness absence for both women and men compared to high strain work in other gender compositions [10]. However, the associations between psychosocial work environment and mental ill-health at workplaces with different types of gender compositions have, to our knowledge, not been studied before. Focusing on gender compositions as part of the psychosocial work environment makes early symptoms of mental ill-health of particular interest as they are likely to be an early sign of an unhealthy environment. Another limitation of previous cross sectional studies is that they fail to take into consideration possible health selection of people with poor health into workplaces with an unhealthy psychosocial work environment. Such health selection could occur due to discrimination of people with poor mental health in the labour market forcing them to accept work at workplaces with an unhealthy psychosocial work environment. As previous mental ill-health is a main predictor of the present mental ill-health [15-17], it is important to further explore such possible health selection mechanisms.

Among the few available studies regarding the associations between psychosocial work environment and gender composition, one study indicated differences in work environment between workplaces with different gender composition, with more job strain for both women and men at workplaces with a majority of women [7]; however, another study showed no significant differences [9]. Work demands, control and support are three major aspects of the psychosocial work environment that have been connected to a variety of negative health outcomes both separately and in combination [18-21]. Although the findings concerning the separate dimensions of job demand, control and social support are not uniform, women often have greater health-related benefits from social support, whereas control is of more importance for men’s health status [22,23]. The divergent results between women and men are often understood and discussed in terms of gender roles [23]. The concept of gender roles has been criticized for not considering that the variation within each gender far exceeds the differences between them [24]. The focus on gender roles also risks hiding the structural context of the stress process [25]. The work environment hypothesis, i.e. that health consequences of the work environment are dependent on the workplace gender compositions, allows us to further explore this structural context as an alternative to dated gender role interpretations. Previous research has also suggested that status variables such as perceiving one’s own work as important [8] and experiencing being devalued by others [26] can be of importance when comparing workplaces with different gender compositions. Both of these aspects, status and being part of a collective purpose, are also identified as latent functions of work [27] that when lacking can result in ill-health [28]. The status variables are related to a societal position rather than a workplace position making them of special interest in relation to the gender segregated labour market.

Our aim was to analyse how workplace gender composition is related to psychological distress and to explore the work environment hypothesis through analysing the importance of the psychosocial work environment for psychological distress at workplaces with different gender compositions.

## Method

The study was performed in Sweden, a country where women and men participate in paid work to almost the same extent. The Swedish labour market is one of the most gender segregated labour markets in the world with only about 13 per cent of workers in occupations where women and men are equally represented [29]. In combination, these two factors make Sweden an interesting setting to study gender composition at workplaces.

### Population

The study was based on the Northern Swedish Cohort that consists of all pupils who did (or should have done) their last year of compulsory school in the town of Luleå in 1981 when most of the participants were 16 years old (506 girls, 577 boys) [30]. In this study, we used data from when the participants were 21 (1986) and 42 (2007) years old. The participation rate of those still alive from the original cohort was 98% in 1986 and 94% in 2007. Only participants who were registered as connected to a workplace in Sweden with more than one employee in 2007 were included in this study (N = 795; 375 women and 420 men) which represents 74% of those still alive in the cohort (N = 1071) and 79% of the participants in 2007 (N =1010). The cohort was investigated at age 16, 18, 30 and 42 years. The first and second data collections were done at the pupils’ schools. For the later follow-ups the participants were invited to reunions at their schools during which they completed the questionnaire. Those who were unable or unwilling to attend the reunions were sent the questionnaire by mail. Participants who did not respond were reminded to do so and structured telephone interviews were conducted with those who preferred and/or had difficulties in reading or writing.

The participants completed an age adapted comprehensive questionnaire developed from validated questions at each follow-up, covering experiences of school/employment environment, socio-economic conditions, self-rated health and health behaviour [30,31]. Data on the gender of all employees at the participants’ workplaces in 2007 were retrieved from Statistics Sweden’s Longitudinal Integration Database for Health Insurance and Labour Market Studies (LISA). From these data the gender composition of the workplace, in which all employees work at the same site (street address), was calculated. The total number of employees at all workplaces was 135 357 and the number of employees at each workplace ranged from 2 to 9130 with a median of 38 employees.

### Measures

#### Exposure variables

The participants’ workplace *gender composition* at age 42 was attained from register data and categorised into three groups: more men (>60 percent men at the workplace), mixed (40–60 percent women at the workplace), and more women (>60 percent women at the workplace).

Psychosocial work environment at age 42 was measured with the Swedish version of the Demand – Control – Support Questionnaire [32]. *Demands* were measured with a five item index. *Control* was measured with four questions on skill discretion and two questions on decision authority. *Support* was measured with a six item index. The four-point option format ranged from ‘agree completely’ to ‘do not agree at all’. The scores in each dimension were added together. The scales were dichotomized at the 75^th^ percentile to identify an exposed group as suggested in previous research [19].

*Being looked down upon* at age 42 was used as an indicator of self-perceived status and measured by the participants’ position in relation to the statement ‘Sometimes it feels like people are looking down on me’. Considering their own work as *important work* at age 42 was used as an indicator of being a part of a collective purpose and was measured with the statement: ‘I am engaged with things that are of importance’. For both statements the respondent could give an answer between 1 (don’t agree at all) and 7 (agree completely). The scales were dichotomized at the 75^th^ percentile.

#### Outcome variable

*Psychological distress at age 42* was measured in the questionnaire with an index consisting of six items (restlessness, concentration problems, worries/nervousness, palpitations, anxiety and other nervous distress) that the participants had experienced during the last 12 months. The questions, derived from the Swedish Survey of Living Conditions [33], are well-known and often used. The index was skewed and therefore dichotomized (0 = no distress, 1 = one or more items of distress). Psychological distress has been shown to be a predictor of later ill-health and mortality for both women and men [34], and the symptoms have high face validity, i.e. it seems possible that such symptoms could be related to psychosocial work environment as well as the gender composition of the workplace.

#### Potential confounders

*Gender* of the cohort participants was entered as a background variable in the logistic regression to ensure that the results were not caused by compositional effects.

*Psychological distress at age 21* was used as an indicator of health related selection. Health related selection implies that earlier psychological distress could affect which workplace a person chooses, i.e. that unhealthy people are selected into workplaces with a specific gender composition. Adjusting for earlier health status is therefore a way to reduce the health-related selection [35]. The questions and dichotomization of the index were exactly the same as at age 42. Age 21 is considered to be appropriate as it is an age before being established in working life.

*Socioeconomic position* at age 42 was measured with the Swedish SEI classification of occupational level [36]. Upper white-collar workers (including self-employed) was used as reference category compared to lower white-collar and blue collar workers.

#### Descriptive variables

*Type of work* at age 42 was measured with a work object classification system based on professions in the Nordic occupational classification [37]. Work was divided into three categories: working with people (e.g. health care, education), working with data (e.g. administration, information technology), and working with things (e.g. manufacturing, construction, cleaning). The Regional Ethical Review Board in Umeå, Sweden, approved this study.

### Analyses

Percentages for psychological distress, exposure variables and background variables in the three gender compositions were calculated for the full sample as well as for women and men separately (Table 1). Differences were analysed with chi-square test. Correlations were tested pairwise with Pearsons test for all exposure variables. There were no correlations > 0.33. Crude and multivariate logistic regressions, with psychological distress at age 42 as outcome, were performed in relation to workplace gender composition (Table 2) calculating odds ratios with 95 per cent confidence intervals. Workplaces with more men were used as reference categories as they represented the lowest prevalence of psychological distress. In Table 3, crude and multivariate regressions with the same outcome were also performed for all work environmental factors adjusting for the gender composition of the workplace. Interaction terms (between gender composition and the work environmental factors) were entered into the analysis in the final model. Interaction between gender composition and gender was also tested (data not shown). It would have been preferable with gender stratified multivariate analyses. However, the limited sample size, with few women and men at workplaces where they were in minority, made this impossible. All statistical analyses were performed using SPSS Statistics 19, and the significance level was at 0.05.

**Table 1:** **Distribution of psychological distress**, **psychosocial work environment factors**, **socioeconomic position and type of work in the three workplace gender compositions** (**percent**)

**Gender composition (age 42)**	**More men**			**Mixed**			**More women**			**Full sample**			**p-values for differences between strata**		
	**all**	**w**	**m**	**all**	**w**	**m**	**all**	**w**	**m**	**all**	**w**	**m**	**all**	**w**	**m**
N	363	65	298	157	78	79	275	232	43	795	375	420			
Psychological distress age 42	25	28	25	42	55*	28*	36	36	37	32	39*	27*	<0.001	0.002	0.22
Psychological distress age 21	25	27	24	24	35*	13*	29	28	33	26	30	23	0.37	0.45	0.03
Change psychological distress between age 21 & 42 (pp)	0†	1	1†	18†	20	15	7†	8†	4	6†	9†	4†			
High demands	30	25	31	32	31	34	30	30	30	30	29	31	0.53	0.69	0.85
Low control	30	33	30	29	36*	21*	32	32	29	31	33	28	0.81	0.80	0.30
Low support	27	21	28	34	28	40	28	27	33	29	26	31	0.24	0.53	0.13
Not important work	24	28	23	26	32	19	14	13	19	21	20	22	0.002	<0.001	0.64
Looked down upon	21	30*	19*	25	26	24	20	20	19	21	23	20	0.45	0.19	0.56
*Socioeconomic position*													<0.001	0.01	<0.001
Upper-white collar	46	55*	44*	67	64	70	53	50*	72*	53	54*	52*			
Lower-white collar	12	28*	8*	18	18	19	17	17*	14*	15	19*	11*			
Blue-collar	42	17*	48*	15	18	11	30	33*	14*	32	27*	37*			
*Type of work*													<0.001	<0.001	<0.001
Working with people	19	25*	18*	26	21	32	70	73	56	38	54*	24*			
Working with data	36	55*	32*	61	64	58	22	20	35	37	35*	38*			
Working with things	45	20*	50*	13	15	10	7	7	9	25	11*	38*			

*Significant differences between women and men within each gender composition (p < 0.05).

†Significant differences in psychological distress between ages 21 and 42 within each gender compositions (p < 0.05).

**Table 2:** **Logistic regression analyses for psychological distress in relation to workplace gender composition** (**ORs and 95**% **CIs**)

	**OR model 0**	**OR model 1**	**OR model 3**
**Gender composition**			
More men (reference)	1	1	1
Mixed	**2.02(1.34 –3.03)**	**1.88(1.20 –2.94)**	**1.77(1.10 –2.83)**
More women	**1.62(1.14 –2.29)**	1.21 (0.78 – 1.89)	1.26 (0.79 – 2.00)

Model 0: Bivariate for each exposure.

Model 1: Adjusted for socioeconomic position, psychological distress at age 21 and gender.

Model 2: Adjusted for socioeconomic position, psychological distress age 21, gender and all psychosocial work environment factors (high demands, low control, low support, not important work and looked down upon).

Bold font indicates significance (p < 0.05).

**Table 3:** **Logistic regression analyses for psychological distress for each work environment exposure** (**ORs and 95**% **CIs**)

	**OR model 0**	**OR model 1**	**OR model 2**	**OR model 3**
**High demands**	**2.25(1.64** – **3.10)**	**2.25(1.61** – **3.15)**	**2.26(1.61** – **3.16)**	**2.14(1.27** – **3.60)**
Gender composition*				
Mixed			**1.93(1.23** – **3.04)**	**1.81(1.04** – **3.16)**
More women			1.25 (0.80 – 1.96)	1.23 (0.74 – 2.06)
Interactionterm demands & gender composition*				
Mixed				1.20 (0.49 –2.93)
More women				1.04 (0.49 –2.21)
**Low control**	**1.89(1.37** – **2.61)**	**1.98(1.40** – **2.81)**	**1.97(1.38** – **2.80)**	**2.23(1.30** – **3.83)**
Gender composition*				
Mixed			**1.92(1.22** – **3.01)**	**2.27(1.34** – **3.84)**
More women			1.24 (0.79 – 1.93)	1.23 (0.73 – 2.07)
Interactionterm control & gender composition*				
Mixed				0.56 (0.23 – 1.40)
More women				0.99 (0.46 – 2.11)
**Low support**	**2.50(1.81** – **3.46)**	**2.66(1.90** – **3.74)**	**2.61(1.85** – **3.66)**	**1.98(1.16** – **3.36)**
Gender composition*				
Mixed			**1.82(1.16** – **2.87)**	**1.82(1.05** – **3.15)**
More women			1.21 (0.78 – 1.90)	0.97 (0.58 – 1.61)
Interactionterm support & gender composition*				
Mixed				1.04 (0.43 – 2.53)
More women				2.10 (0.96 – 4.57)
**Looked down upon**	**2.27(1.59** – **3.24)**	**2.20(1.52** – **3.20)**	**2.15(1.47** – **3.13)**	1.55 (0.86 – 2.77)
Gender composition*				
Mixed			**1.87(1.20** – **2.94)**	1.53 (0.90 – 2.58)
More women			1.32 (0.85 – 2.05)	1.17 (0.71 – 1.90)
Interactionterm looked down upon & gender composition*				
Mixed				2.14 (0.80 –5.68)
More women				1.55 (0.66 – 3.63)
**Not important work**	**1.65(1.15** – **2.36)**	**1.71(1.18** – **2.49)**	**1.73(1.18** – **2.52)**	1.62 (0.93 – 2.81)
Gender composition*				
Mixed			**1.90(1.21** – **2.98)**	**1.95(1.17** – **3.28)**
More women			1.37 (0.88 – 2.13)	1.28 (0.79 – 2.08)
Interactionterm important work & gender composition*				
Mixed				0.89 (0.35 – 2.29)
More women				1.40 (0.56 – 3.46)

*More men reference.

Model 0: Bivariate for each exposure.

Model 1: Adjusted for gender, socioeconomic position, psychological distress age 21.

Model 2: Adjusted for gender, socioeconomic position, psychological distress age 21, gender composition.

Model 3: Adjusted for gender, socioeconomic position, psychological distress age 21, gender composition, interaction terms for gender composition and exposure.

Bold font indicates significance (p < 0.05).

## Results

The prevalence of psychological distress differed on the basis of workplace gender composition in the total population as presented in Table 1. The highest prevalence of psychological distress at age 42 was found among women at workplaces with a mixed gender composition, whereas workplaces with more men had the lowest prevalence among both women and in the total population. In the gender stratified descriptive analyses, significant differences between workplace gender composition and psychological distress was found for women but not for men at age 42 (Table 1). At age 21 significant differences in psychological distress was found among men but not among women or in the total population. The prevalence of psychological distress increased considerably between ages 21 and 42 in the full sample of participants who at age 42 worked at mixed workplaces (though men started out and remained at a significantly lower prevalence compared to women), increased moderately among participants at workplaces with more women and remained stable among participants at workplaces with more men.

The psychosocial work environment was rather similar in the three gender compositions; the only variable that differed significantly between the gender compositions was the status variable “not important work” (Table 1). Participants at workplaces with more women were most likely to describe their work as important. The gender composition was also related to socioeconomic position, with a higher proportion of upper-white collar participants at workplaces with a mixed gender composition, whereas blue collar workers were more common among men at workplaces with more men and among women at workplaces with more women. Working with people (for example, health care and education) was the most common type of work at workplaces with more women, and working with data (for example different types of office work) was most common at mixed workplaces. At workplaces with a majority of men, women typically worked with data, whereas men typically worked with things (for example manufacturing). Comparing women and men within each gender composition, there were only two significant differences regarding the psychosocial work environment: a larger proportion of women reported low control at the mixed workplaces and being looked down upon at workplaces with more men. At workplaces with more men there were also significant differences between women and men in socioeconomic position and type of work.

The logistic regression (Table 2) confirmed associations between gender composition and psychological distress. Employees at workplaces with a mixed gender composition also remained at a higher odds ratio of psychological distress after all adjustments (socioeconomic position, psychological distress at age 21, all psychosocial work environment factors and gender) compared to employees at workplaces with more men. Employees at workplaces with more women did not have a higher OR of psychological distress compared to workplaces with more men when the association was adjusted for covariates in model 1, and among the covariates it was the adjustment for gender that made the association insignificant.

Table 3 provides the results from the multivariate logistic regression analysis for psychological distress in relation to each of the work environmental factors. All work environmental factors were associated with psychological distress in the crude analyses as well as in model 1 and 2. Mixed workplaces also had a higher OR for psychological distress compared to workplaces with more men for all work environmental factors. No significant interactions were found in any model. The results indicate that there were no differences in the association between work environmental factors and psychological distress according to gender composition. The association between gender and psychological distress was also analysed with corresponding analysis (data not shown). The association between gender and psychological distress turned insignificant when the interaction term for gender composition and gender was introduced in the model and no interaction was found.

## Discussion

Working at a workplace with a mixed gender composition was related to a higher likelihood of psychological distress compared to workplaces with more men, even after adjustment for socioeconomic position, psychological distress at age 21, psychosocial work environment factors and gender. Contextual perceptions of the psychosocial work environment based on the workplace gender composition could not explain the relations.

Our findings regarding worse health at mixed workplaces are interesting because they contradict previous research on the occupational level that suggests better health in those few occupations that are gender-integrated [7]. A possible explanation could be that gender composition is measured on different levels – on occupational and on workplace level, and that gender integrated occupations means something different compared to mixed workplaces. Previous research also suggests that women at workplaces with more women are worse off in terms of mental health [8]. In our study the differences in psychological distress between workplaces with more women and workplaces with more men are not significant after adjusting for gender. Our results indicate that the higher likelihood of psychological distress at workplaces with more women in the crude analysis is explained by a compositional effect, i.e. that there are more women at these workplaces, and women as a group have a higher prevalence of psychological distress. However, these results must be interpreted with caution as there are few men at these workplaces and there might not be enough statistical power to detect an association. The descriptive analyses indicate that the prevalence of psychological distress for men at workplaces with more women is at the same level as for women.

Concerning the second part of our aim exploring the work environment hypothesis, our results show that the differences in psychological distress between the three gender compositions could not be explained by differences in their perception of the psychosocial work environment, and consequently the work environment hypothesis is not supported. Previous research has suggested an influence of gender composition on the association between high strain jobs and sickness absence for both women and men [10]. Our results indicate that when the outcome is self-reported psychological distress rather than sickness absence, gender composition does not influence the association between the psychosocial work environment and the outcome in a corresponding way. As the explanation to differences in prevalence of psychological distress between gender compositions does not seem to be found in the psychosocial work environment, more research is needed to explore other possible explanations. For the psychosocial work environment factors included in this study, only one factor (not important work) differed significantly in prevalence between workplace gender compositions. Previous research also indicates that workplaces with different gender composition do not differ significantly in the reported character of their psychosocial work environment [9]. These findings indicate that the explanation to differences in health connected to workplace gender composition might be found elsewhere. One possible way forward could be to explore how workplace cultures regarding health behaviour fits into this picture. However, although the psychosocial work environment cannot explain the differences in psychological distress between the gender compositions, our study confirms that demand, control and support are all related to mental distress at all types of workplaces. Our study also adds the status variables such as important work and being looked down upon as factors that can be of interest to explore in future research.

The longitudinal design allows us to reflect on the development of psychological distress prevalence over time. The lowest prevalence of psychological distress at age 21 was found among men who at age 42 worked at mixed workplaces; but at age 42 the differences compared to men at workplaces with other gender compositions were no longer significant. These results suggest that there might be a positive health selection of men into the mixed workplaces, i.e. that the group of men who at age 42 worked at mixed workplaces started their working life with lower prevalence of psychological distress compared to men who ended up at workplaces with other gender compositions. We can also see that there might be a negative health selection of men into workplaces with more women. The lack of significant differences in psychological distress between gender compositions for men at age 42 is consistent with several previous studies on the occupational [12] and workplace level [7,14]. However, the new finding of a potential positive health selection of men into the mixed workplaces and negative health selection of men into workplaces with more women requires more attention in future studies. The longitudinal design also indicate that the health differences between the gender compositions might evolve during working life (between ages 21 and 42). At workplaces with a mixed gender composition the prevalence of psychological distress increased considerably during working life for both women and men, but women start and end up at a higher prevalence compared to the men. This suggests that the conditions in working life might have a negative influence on mental health for both women and men at mixed workplaces, and that the differences that were present at an earlier age have been scaled up. It is possible that the work environment is similar for women and men at these workplaces and that initial differences in mental ill-health explain the differences at age 42. It is also possible that the workplaces are not really gender integrated, but that women and men have very different work situations with diverse associations with mental ill-health. The latter interpretation is to some extent supported in the descriptive analysis where more women than men at these workplaces reported low control. Also, previous analyses on the same population showed that a mixed gender composition often co-existed with gender inequalities in salaries, educational levels and parental leave at the workplace [38]. Regardless of the explanation to the differences in prevalence between women and men, the similar development of psychological distress over time for women and men in the same gender composition gives some support for the assumption that the gendered organizations are of importance for mental ill-health, and perhaps more so than gender roles attached to the individual, which was suggested previously [22], [23]. However, more research on larger samples is needed to clarify the importance of gender at different levels.

### On the method

A major strength of the present study is the high quality of data from a longitudinal cohort with high response rate. The longitudinal cohort design makes it possible to adjust for previous psychological distress, which has not been possible in previous cross-sectional studies [8,9]. The use of register data for determining gender composition of the workplace also ensures high validity compared to previous studies that have mainly relied on self-reported gender composition. A limitation of the study is that the relatively small sample size makes separate analyses for women and men impossible in the logistic regression. In future research, gender stratified analyses should be used to further explore if men and women at workplaces with various gender compositions are similarly affected [39]. Future research is also needed to analyse the importance of factors in unpaid work, such as family responsibility, in relation to gender composition of workplaces and psychological distress. Another limitation is that both exposures (work environment factors) and the outcome (psychological distress) are self-reported. However, although it is possible that respondents with poor mental health also report worse psychosocial work environment, there is little reason to believe that such “over reporting” would differ between the gender compositions which is the main focus of this study. Both severe and less severe complaints of psychological distress have been shown to be related to later ill-health and all-cause mortality [34].

In our study we have chosen to include demand, control and support as separate dimensions of the psychosocial work environment rather than combining them into job strain and iso-strain. It has been debated whether to use the variables in combination or as separate dimensions, and especially in longitudinal studies the support for the combinations in relation to psychological wellbeing has been scarce [19,20]. In empirical testing of the demand-control-support model, many studies have also found differences between women and men with more support for using the high strain combination on populations of men [20]. It has also been suggested that men, to a higher degree, work in organisations and occupations where they have more possibilities to influence their work situation, although this is not always caught by the control measure. This suggestion was supported by an externally assessed validation of the demand-control model in which women who described their work as active did not have more creative tasks than women in high–strain or passive jobs [40].

The Northern Swedish Cohort is a homogenous group in terms of age and geographic location, although 41 per cent lived elsewhere than Luleå at the latest follow up. However, the labour market structure of Luleå is representative of Sweden as a whole in regards to the distribution between branches of business in 2007 at the latest follow up [41], and the cohort has also proven to be comparable to the country as a whole with regard to socio-demographic factors as well as health status and health behaviour [30]. The sample in the present study is comparable to Sweden regarding socioeconomic position in the age group between 25 and 44 years [42]. The workplace sample (all employees at the participants’ workplaces that were used to calculate the gender composition exposure) has a similar age structure as the general population in Sweden, but a higher educational level [42]. The generalization of the results may be limited to similar age groups and workplaces as well as to other countries with similar gender segregated labour market and high labour market participation for both women and men.

## Conclusions

In contrast to previous research this study suggests that employees at workplaces with a mixed gender composition might have a higher likelihood of psychological distress compared to workplaces with more men. This association cannot be explained by differences in the perception of the psychosocial work environment, and thus the work environment hypothesis is not supported. Workplaces with a mixed gender composition needs further research attention to explain the negative development of psychological distress during working life for both women and men at these workplaces.

## Competing interests

The authors declare that they have no competing interests.

## Authors’ contributions

All authors participated in the design of the paper and contributed to the analyses of the data. SE drafted the manuscript. All authors read and approved the final manuscript.

## Pre-publication history

The pre-publication history for this paper can be accessed here:

http://www.biomedcentral.com/1471-2458/14/241/prepub

## References

[B1] Messing K Östlin P Gender equality, work and health: A review of the evidence. In 2006 Geneva: World Health Organisation

[B2] Statistics Sweden Women and men in Sweden 2010 Örebro: SCB-tryck

[B3] Connell R Glass ceiling or gendered institutions? Mapping the gender regimes of public sector worksites Public Adm Rev 2006 66 6 837 849 10.1111/j.1540-6210.2006.00652.x

[B4] Evans O Steptoe A The contribution of gender-role orientation, work factors and home stressors to psychological well-being and sickness absence in male- and female-dominated occupational groups Soc Sci Med 2002 54 481 492 10.1016/S0277-9536(01)00044-2 11848269

[B5] Hall EM Gender, work control, and stress: a theoretical discussion and an empirical test Int J Health Serv 1989 19 4 725 745 10.2190/5MYW-PGP9-4M72-TPXF 2583884

[B6] Savikko A Lanne M Spak F Hensing G No higher risk of problem drinking or mental illness for women in male-dominated occupations Subst Use Misuse 2008 43 8–9 1151 1169 18649236 10.1080/10826080801917918

[B7] Bryngelson A Bacchus Hertzman J Fritzell J The relationship between gender segregation in the workplace and long-term sickness absence in Sweden Scand J Public Health 2011 39 6 618 626 10.1177/1403494811414242 21733963

[B8] Hensing G Alexanderson K The association between sex segregation, working conditions, and sickness absence among employed women Occup Environ Med 2004 61 2 e7 10.1136/oem.2002.005504 14739391 PMC1740719

[B9] Svedberg P Bildt C Lindelöw M Alexanderson K Self-reported health among employees in relation to sex segregation at work sites J Occup Health 2009 51 3 223 231 10.1539/joh.L8033 19336968

[B10] Jonsson R Lidwall U Holmgren K Does unbalanced gender composition at the work place influence the association between psychosocial working conditions and sickness absence? Work 2013 46 1 59 66 23241702 10.3233/WOR-121529

[B11] The Swedish Social Insurance Agency Sick leave diagnosis in different occupations [In Swedish: Sjukskrivningsdiagnoser i olika yrken] Social Insurance Report 2011:17 In: http://www.forsakringskassan.se/wps/wcm/connect/a8df6885-8608-42b9-9483-9e910f88077e/socialforsakringsrapport_2011_17.pdf?MOD=AJPERES

[B12] Hunt K Emslie C Orth-Gomér K, Chesney M, Wenger NK Men's work, women's work? Occupational sex ratios and health Women, Stress and Heart Disease. edn 1998 Mahwah, NJ: Lawrence Erlbaum Associates 87 107

[B13] Kanter RM Men and women of the corporation 1977 New York: Basic Books

[B14] Mastekaasa A Sickness absence in female- and male-dominated occupations and workplaces Soc Sci Med 2005 60 10 2261 2272 10.1016/j.socscimed.2004.10.003 15748674

[B15] Fergusson DM Horwood LJ Ridder EM Beautrais AL Subthreshold depression in adolescence and mental health outcomes in adulthood Arch Gen Psychiatry 2005 62 1 66 72 10.1001/archpsyc.62.1.66 15630074

[B16] Fichter MM Kohlboeck G Quadflieg N Wyschkon A Esser G From childhood to adult age: 18-year longitudinal results and prediction of the course of mental disorders in the community Soc Psychiatry Psychiatr Epidemiol 2009 44 9 792 803 10.1007/s00127-009-0501-y 19212695

[B17] Jonsson U Bohman H von Knorring L Olsson G Paaren A von Knorring AL Mental health outcome of long-term and episodic adolescent depression: 15-year follow-up of a community sample J Affect Disord 2011 130 3 395 404 10.1016/j.jad.2010.10.046 21112639

[B18] Karasek RA Theorell T Healthy work: Stress, productivity and reconstruction of working life 1990 New York: Basic Books

[B19] de Lange AH Taris TW Kompier MA Houtman IL Bongers PM The very best of the millennium”: longitudinal research and the demand-control-(support) model J Occup Health Psychol 2003 8 4 282 305 14570524 10.1037/1076-8998.8.4.282

[B20] Van der Doef M Maes S The Job Demand-Control (-Support) model and psychological well-being: a review of 20 years of empirical research Work Stress 1999 13 2 87 114 10.1080/026783799296084

[B21] Kivimäki M Nyberg ST Batty GD Fransson EI Heikkilä K Alfredsson L Bjorner JB Borritz M Burr H Casini A Clays E De Bacquer D Dragano N Ferrie JE Geuskens GA Goldberg M Hamer M Hooftman WE Houtman IL Joensuu M Jokela M Kittel F Knutsson A Koskenvuo M Koskinen A Kouvonen A Kumari M Madsen IE Marmot MG Nielsen ML Job strain as a risk factor for coronary heart disease: a collaborative meta-analysis of individual participant data Lancet 2012 380 9852 1491 1497 10.1016/S0140-6736(12)60994-5 22981903 PMC3486012

[B22] Beehr TA Farmer SJ Glazer S Gudanowski DM Nair VN The enigma of social support and occupational stress: source congruence and gender role effects J Occup Health Psychol 2003 8 3 220 231 12872959 10.1037/1076-8998.8.3.220

[B23] Gadinger MC Fischer JE Schneider S Terris DD Kruckeberg K Yamamoto S Frank G Kromm W Gender moderates the health-effects of job strain in managers Int Arch Occup Environ Health 2010 83 5 531 541 10.1007/s00420-009-0477-7 19888594

[B24] Connell RW Gender 2002 Cambridge: Polity Press

[B25] Pearlin LI The sociological study of stress J Health Soc Behav 1989 30 3 241 256 10.2307/2136956 2674272

[B26] Elwér S Aléx L Hammarström A Health against the odds: experiences of employees in elder care from a gender perspective Qual Health Res 2010 20 9 1202 1212 10.1177/1049732310371624 20519432

[B27] Jahoda M Employment and unemployment: A social psychological analysis 1982 New York: Cambridge University Press

[B28] Janlert U Hammarström A Which theory is best? Explanatory models of the relationship between unemployment and health BMC Public Health 2009 9 235 10.1186/1471-2458-9-235 19602230 PMC2720386

[B29] Statistics Sweden Women and men in Sweden 2012 Örebro: SCB-tryck

[B30] Hammarström A Janlert U Cohort Profile: The Northern Swedish Cohort Int J Epidemiol 2012 41 6 1545 1552 10.1093/ije/dyr118 21828110

[B31] Hammarström A Youth unemployment and ill-health (In Swedish) 1986 Karolinska Institute: Stockholm

[B32] Landsbergis P Theorell T Schwartz J Greiner BA Krause N Measurement of psychosocial workplace exposure variables Occup Med 2000 15 1 163 188 10620790

[B33] Sweden S Swedish survey of living conditions. In 1980 Statistics Sweden: Stockholm

[B34] Ringbäck Weitoft G Rosen M Is perceived nervousness and anxiety a predictor of premature mortality and severe morbidity? A longitudinal follow up of the Swedish survey of living conditions J Epidemiol Community Health 2005 59 9 794 798 10.1136/jech.2005.033076 16100319 PMC1733128

[B35] Artazcoz L Borrell C Cortes I Escriba-Aguir V Cascant L Occupational epidemiology and work related inequalities in health: a gender perspective for two complementary approaches to work and health research J Epidemiol Community Health 2007 61 Suppl 2 ii39 ii45 18000116 10.1136/jech.2007.059774 PMC2465767

[B36] Statistics Sweden Socioekonomisk indelning (SEI) [Swedish socioeconomic classification] Meddelanden i samordningsfrågor 1982 1982:4 Stockholm: Statistics Sweden

[B37] Härenstam A Karlqvist L Bodin L Nise G Scheele P The Moa Research Group: Patterns of working and living conditions: a holistic, multivariate approach to occupational health studies Work Stress 2003 17 1 73 92 10.1080/0267837031000099168

[B38] Elwér S Harryson L Bolin M Hammarström A Patterns of gender equality at workplaces and psychological distress PloS one 2013 8 1 e53246 10.1371/journal.pone.0053246 23326404 PMC3541387

[B39] Messing K Stock SR Tissot F Should studies of risk factors for musculoskeletal disorders be stratified by gender? Lessons from the 1998 Quebec Health and Social Survey Scand J Work Environ Health 2009 35 2 96 112 10.5271/sjweh.1310 19305934

[B40] Waldenström K Härenstam A Does the job demand-control model correspond to externally assessed demands and control for both women and men? Scand J Public Health 2008 36 3 242 249 10.1177/1403494807085079 18519292

[B41] Elwér S Gender equality and health experience: Workplace patterns in Northern Sweden 2013 Umeå: Umeå University

[B42] Statistics Sweden Living Conditions Surveys 2013 In. http://www.scb.se

